# Mating Assay: Plating Below a Cell Density Threshold is Required for Unbiased Estimation of Plasmid Conjugation Frequency of RP4 Transfer Between *E. coli* Strains

**DOI:** 10.1007/s00248-024-02427-7

**Published:** 2024-08-28

**Authors:** Zhiming He, Barth F. Smets, Arnaud Dechesne

**Affiliations:** 1 Department of Biotechnology and Biomedicine, Technical University of Denmark, Søltofts Plads Building 221, 2800 Kgs. Lyngby, Denmark; 2 Sino-Danish College (SDC) for Education and Research, University of Chinese Academy of Sciences, 8000 Aarhus C, Denmark; 3 Department of Biological and Chemical Engineering – Environmental Engineering, Aarhus University, Ole Worms Allé 3, 8000 Aarhus C, Denmark

**Keywords:** Conjugation, Selective plating, Plate counting, Enumeration, Guideline, Plasmid

## Abstract

Mating assays are common laboratory experiments for measuring the conjugation frequency, i.e. efficiency at which a plasmid transfers from a population of donor cells to a population of recipient cells. Selective plating remains a widely used quantification method to enumerate transconjugants at the end of such assays. However, conjugation frequencies may be inaccurately estimated because plasmid transfer can occur on transconjugant-selective plates rather than only during the intended mating duration. We investigated the influence of cell density on this phenomenon. We conducted mating experiments with IncPα plasmid RP4 harbored in *Escherichia coli* at a fixed cell density and mating conditions, inoculated a serial dilution of the mating mixture on transconjugant-selective plates or in transconjugant-selective broth, and compared the results to a model of cell-to-cell distance distribution. Our findings suggest that irrespective of the mating mode (liquid vs solid), the enumeration of transconjugants becomes significantly biased if the plated cell density exceeds 28 Colony Forming Unit (CFU)/mm^2^ (or 1.68•10^5^ CFU/standard 9 cm Petri dish). This threshold is determined with a 95% confidence interval of ± 4 CFU/mm^2^ (± 2.46•10^4^ CFU/standard 9 cm Petri dish). Liquid mating assays were more sensitive to this bias because the conjugation frequency of RP4 is several orders of magnitude lower in suspension compared to surface mating. Therefore, if selective plating is used, we recommend to plate at this density threshold and that negative controls are performed where donors and recipients are briefly mixed before plating at the same dilutions as for the actual mating assay. As an alternative, a liquid enumeration method can be utilized to increase the signal-to-noise ratio and allow for more accurate enumeration of transconjugants.

**Supplementary Information:**

The online version contains supplementary material available at 10.1007/s00248-024-02427-7.

## Introduction

Conjugation is a phenomenon that facilitates the exchange of genetic information between microbes within a microbial community [1, 2]. It involves the mobilization of genetic elements such as plasmids [3], and integrative and conjugative elements [4, 5].

Understanding the drivers and barriers of conjugation is essential for predicting or hindering the spread of virulence factors and antibiotic resistance genes [6, 7] and facilitating traits such as biotransformation functions [8, 9]. Therefore, it is useful to employ mating assays, where a population of donors transfer plasmids to a population of recipients to estimate the conjugation frequency and evaluate the efficiency of the plasmid transfer. Efficiency is defined as the number of recipients that have received a plasmid (i.e. transconjugants) at the end of a mating experiment relative to the number of donors or recipients, or both at the beginning of the mating assay [10].

Typically, mating assays are conducted with pure cultures of donors and recipients [11–15] with mating occuring either in a liquid suspension [11, 12, 14, 16–18] or on a solid surface [15, 19–23] because different classes of plasmids transfer more efficiently in one or the other condition, depending on the type of pilus the plasmid encodes [24]. At the end of the mating duration, the mating mixture is often plated on agar plates with appropriate selective agents (e.g. antibiotics) to enumerate transconjugants, donors, and recipients after sufficiently long incubation [11, 13, 25–28].

**Fig. 1:**
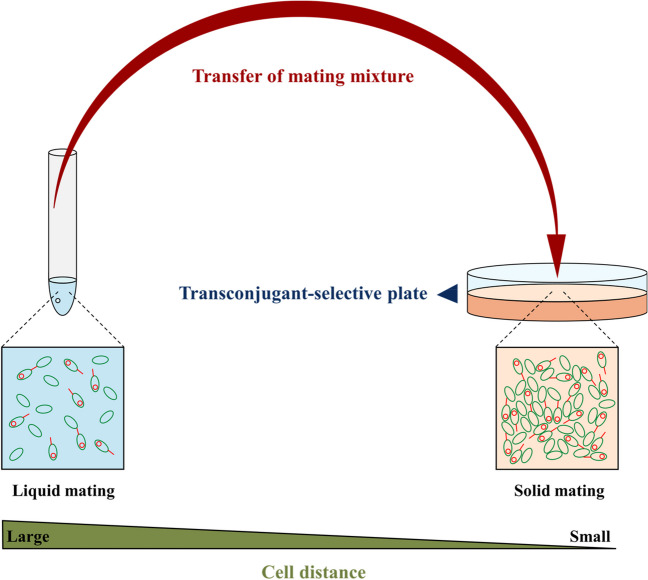
Illustration of the phenomenon of plasmid transfer taking place on the transconjugants-selective plates after mating in liquid suspension

However, it is essential to acknowledge that experimental bias can arise from this enumeration technique, as many antibiotics seldom kill or inhibit growth of bacteria immediately [29, 30]. Although antibiotics may halt cell division within minutes, cells can remain metabolically active under physiological stress [31]. This phenomenon can provide opportunities for donor cells to transfer a plasmid to recipients on the transconjugant-selective plates if they have been plated at high density (i.e. in close proximity) and thus artefactually inflate the number of transconjugants (Fig. 1).

This consequently impedes the accurate estimation of plasmid transfer events under the initial mating conditions. The probability of such overestimation increases with increasing density of parent cells plated on the transconjugant-selective plates [32]. Although this issue of plasmid transfer occurring on transconjugant-selective plates has long been recognized [21, 33], and even mentioned as one of the assumptions to consider in appropriate guidelines [34], an examination of recent literature [11–13, 16, 17, 26–28, 35–38] shows that this phenomenon has neither been explicitly reported nor has it been regularly taken into consideration.

In this study, we sought to determine the influence of cell densities on plasmid transfer by disentangling the number of transconjugants produced on transconjugant-selective plates relative to those produced during the mating assay, conducted either in liquid suspension or on a solid surface. Hereby, we offer additional insight into this phenomenon and underscore the significance of cell densities for the accurate estimation of conjugation frequency.

## Methods

### Bacterial Strains and Conjugative Plasmid

To determine how plasmid transfer occurring on transconjugant-selective plates is influenced by cell densities, we used model strains *Escherichia coli* (*E. coli*) MG1655 as donors and recipients along with the IncPα model conjugative plasmid RP4 (Table S1). To prepare for the mating assay, we inoculated the *E. coli* MG1655 donor carrying the RP4 plasmid from a freshly streaked plate into 10 mL Luria–Bertani (LB) medium with tetracycline at 20 µg/mL and ampicillin at 100 µg/mL. The *E. coli* MG1655 recipient with chromosomal resistances to kanamycin, nalidixic acid, and rifampicin was grown in the same medium without antibiotics. Both strains were kindly provided by Professor Søren Sørensen from the Department of Biology at the University of Copenhagen.

### Preparation of Donor and Recipient Culture

After growth for 16 h at 30 °C, the cultures were harvested by centrifugation at 4000 RCF for 5 min at room temperature and washed twice in phosphate-buffered saline (PBS). After washing, the cultures were resuspended in PBS and diluted to an OD600 of 0.5 to obtain an initial cell density of 10^8^ CFU/mL (Table S2).

### Liquid Mating Followed by Solid or Liquid Enumeration Method

After strain preparation**,** the donor and recipient cultures were mixed in glass tubes at a 1:1 ratio (2 mL total) with a final glucose concentration of 0.4%, and incubated at 28˚C for 0, 60, and 480 min under shaking conditions. To interrupt mating, the tubes were cooled down for 1 min on ice, thoroughly vortexed for 1 min, and kept on ice until further processing. A serial dilution series of the mating mixtures (10^0^–10^−3^) were plated on transconjugant-selective plates (9 cm Petri dish, 6082 mm^2^) containing 20 µg/mL tetracycline, 100 µg/mL ampicillin, and 50 µg/mL nalidixic acid (Table S3). The quantification method of T/DR was used, where T, D, and R denote the density of transconjugants, donors, recipients, respectively (Table S4). Enumeration of donors and recipients were done by spotting several aliquots of 10 µL of a serial dilution (10^–3^-10^–6^) on donor-selective plates containing 20 µg/mL tetracycline and 100 µg/mL ampicillin and on recipient-selective plates containing 50 µg/mL nalidixic acid. The expected selection outcome can be found in the supplementary information (Table S5). Additionally, liquid mating was repeated where transconjugants, donors and recipients were enumerated by using a most-probable number liquid enumeration method (Fig. S1). After mating, 10 µL of a one-step dilution series of mating mixture (10^0^–10^−1^) was transferred to a 96-well microplate (48 wells for each replicate) containing 190 µL LB broth with the same selective antibiotics as described above. For donor and recipient enumeration, 20 µL of the mating mixture was serially diluted (10^–1^-10^–9^) in a 96-well microplate. Plates were incubated overnight at 37˚C and inspected for observable growth.

### Solid Mating Followed by Solid Enumeration Method

For solid mating, sterile 25 mm Whatman® glass microfiber filters Grade GF/C (WHA1822025) were placed onto each chamber of a 1225 Millipore Sampling Manifold (XX2702550) and pre-wetted with PBS followed by placing sterile 25 mm Whatman® Cyclopore® polycarbonate filters (WHA70632502) with working area of 280 mm^2^ on top of the glass microfiber filters. Next, 2 mL of PBS was added to all chambers to flush the filters. Following strain preparation, the donor and recipient cultures were added to each chamber in a 1:1 ratio (2 mL in total) and filtered. Once filtered, the polycarbonate filters were transferred to nutrient-free PBS agar plates containing 0.4% glucose to allow for solid mating and incubated at 28˚C for 0, 60, and 480 min. After mating, the polycarbonate filters were transferred to 15 mL falcon tubes containing 2 mL PBS, subsequently vortexed vigorously for 1 min to detach cells, and kept on ice until further processing. The quantification method of T/DR, as described in Section "Liquid Mating Followed by Solid or Liquid Enumeration Method.", was used (Table S4). To enumerate transconjugants, donors, and recipients, cells were serially diluted (10^0^–10^−5^ for transconjugants and 10^3^–10^−6^ for donors and recipients), plated or spotted, and enumerated in a similar manner as in Section "Liquid Mating Followed by Solid or Liquid Enumeration Method." (Table S5).

### Estimating Cell-to-cell Distances using a Distribution Function

The distribution of cells on a surface can be described as a homogenous Poisson point process. The distance to the closest cell (r) has a distribution function F(r) = $1-{e}^{-\lambda {r}^{2}}$ with λ representing the cell density as previously described [39]. We assumed a distance of 1 µm or less between cells as an indication of a successful donor-recipient mating pair. The calculations were done using R (version 4.3.2). To generate illustrations of donor and recipient cell distributions, we used the rpois() function to generate a Poisson-distributed number of points representing donor and recipient cells, which were randomly placed across space by generating random coordinates. To verify that the simulated patterns complied with the expected distance distribution, the pairwise distances between cells were calculated using the dist() function.

### Statistical Analysis

Significant differences were investigated using independent samples t-test. P-values of < 0.025, < 0.01, and < 0.001 were considered significant, with increasing levels of significance. The cutoff for significance at *p* < 0.025 was determined using the Bonferroni correction, calculated as α_n_ = α_o_/n, where α_o_ is the significance level of 0.05 and n is the number of groups being compared. The 95% confidence intervals were estimated by pooling the standard deviations of the donor and recipient cell densities.

## Results

### Artefactual Transconjugants are Generated when a Cell Density Threshold is Exceeded on Transconjugant-Selective Plates

**Fig. 2:**
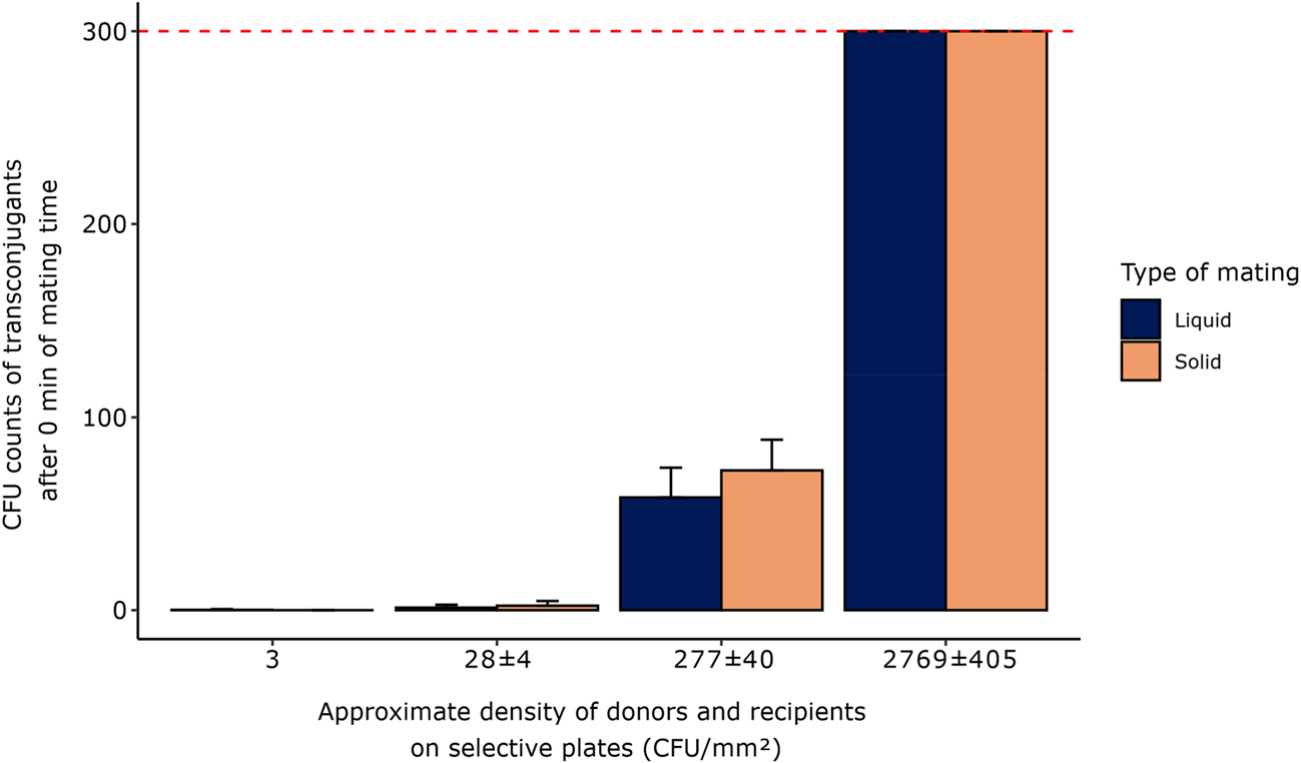
Average number of transconjugants enumerated on transconjugant-selective plates after plating mixtures of donors and recipients at different densities. The mixtures were either plated directly after mixing donors and recipients in liquid suspension or after adding donors and recipients onto a filter before detaching and resuspending the cells in liquid. The cell densities are estimated with a 95% confidence interval. The red dashed line shows the upper limit of quantification at > 300 CFUs. Error bars represent standard deviations. Number of replicates for transconjugants, donors and recipients were n = 3, 21, and 23, respectively

Plating the mating mixture immediately after mixing donor and recipients in the mating assays confirmed that RP4 transfer between *E. coli* strains could readily occur on transconjugant-selective plates, provided the cell density was high enough. The number of cells on the transconjugant-selective plates were determined by taking the average of the donor and recipient cell density (Fig. 2, Tables S2, S6-S9).

When plating a mixture of donors and recipients on the transconjugant-selective plates at a high cell density (≥ 277 CFU/mm^2^ or 1.68•10^6^ CFU/standard 9 cm Petri dish with a 95% confidence interval of ± 40 CFU/mm^2^ or ± 2.46•10^5^ CFU/standard 9 cm Petri dish), many transconjugant colonies were observed. In contrast, at low cell density (≤ 28 ± 4 CFU/mm^2^ or 1.68•10^5^ ± 2.46•10^4^ CFU/standard 9 cm Petri dish), very few or no transconjugants were observed. In conclusion, plating a high density of donors and recipients from the mating mixture to enumerate transconjugants will lead to artefactual inflation of the transconjugant numbers. Consequently, the outcome of an experiment studying the efficiency of plasmid transfer may not effectively address the initial research question.

**Fig. 3:**
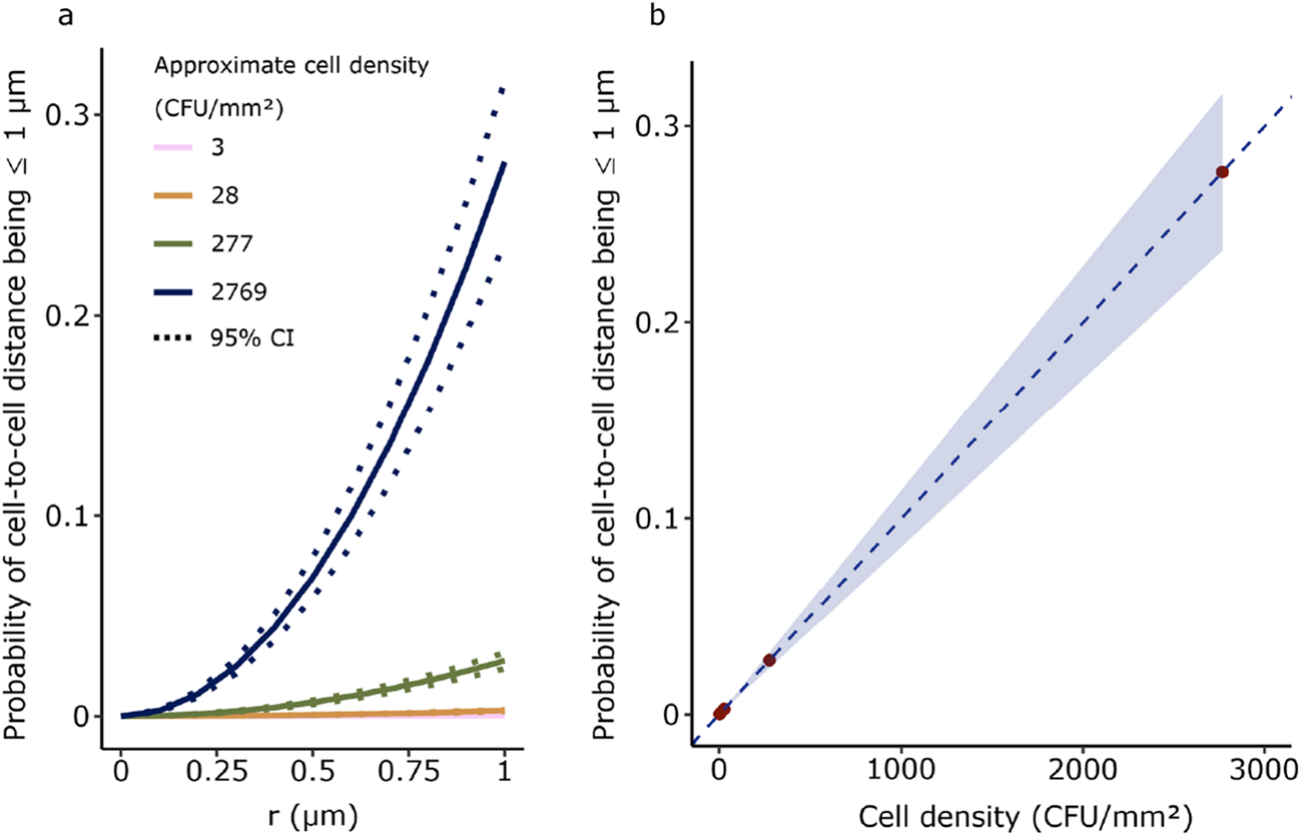
**a** The distribution function shows the probability for a cell to have at least one neighboring cell within a circle of radius (r) at different cell densities of 3 (pink), 28 (orange), 277 (green), and 2769 (dark blue) CFU/mm^2^ with the dotted curves representing the 95% confidence interval at ± 4 (orange), ± 40 (green), and ± 405 (dark blue). **b** The probability of cells to nearest neighbor distances being equal to or less than 1 µm as a function of cell density. The dark red dots represent the cell densities in (a) with the shaded area showing the 95% confidence interval of the probability, calculated from the upper and lower limit of cell densities

Next, we further investigated these cell densities (Fig. 2) to see if the link between cell density and the formation of transconjugants could be captured with a simple model describing the homogenous Poisson point process as presented in Section "Estimating Cell-to-cell Distances using Distribution Function". Assuming a homogenous Poisson point process, the probability for a cell to be at a distance of 1 µm or less to its nearest neighbor is 0.00028 ± 0.00004%, 0.0028 ± 0.0004%, 0.028 ± 0.004%, and 0.28 ± 0.04% for cell densities of 3, 28 ± 4, 277 ± 40, and 2769 ± 405 CFU/mm^2^, respectively (Fig. 3a and b, Figs. S2 and S3).

We then multiplied the probability by the number of cells per plate, to estimate the expected number of transconjugants generated from RP4 transfer on transconjugant-selective plates. Using the estimated probabilities of individual cells being within a distance of 1 µm or less for each cell density, the expected transconjugant counts were calculated to be < 1, 5, 466 ± 34, and 46554 ± 3442 CFU. These estimates correspond to total donor and recipient numbers of 1.68•10^4^ ± 2.46•10^3^, 1.68•10^5^ ± 2.46•10^4^, 1.68•10^6^ ± 2.46•10^5^, and 1.68•10^7^ ± 2.46•10^6^ CFU/standard 9 cm Petri dish, respectively. While the expected number of transconjugants when plating 10^4^ and 10^5^ CFU/standard 9 cm Petri dish is consistent with our observations on transconjugant-selective plates, the expected number of transconjugants when plating 10^6^ CFU/standard 9 cm Petri dish is higher than observed experimentally (Fig. 3, Tables S6-S9). Overall, this indicates a high conjugation efficiency on the transconjugant-selective plates when donors and recipients are in close proximity, although there may be saturation of mating pairs at very high densities. Thus, the distribution function can be leveraged to determine the optimal cell density for plating, based on the risk one is willing to take to have a certain number of artefactual transconjugants on the transconjugant-selective plates.

The permissible number of transconjugants at each density will depend on the efficiency of plasmid transfer during the mating assay and its duration. Furthermore, this model, potentially adjusted with data from the relevant plasmids or strains, can be applied to correct datasets where plating was used as enumeration method for artefactual plasmid transfer, such as in published studies.

### Effect of Experimental Design (Mating Mode and Enumeration Method) on Signal-to-noise Ratio for RP4 Conjugation Frequency Estimations

Knowing that the cell density on the transconjugant-selective plates should not exceed 28 ± 4 CFU/mm^2^ for RP4 transfer between *E. coli* strains, we explored if liquid or solid mating assays can generate enough transconjugants (signal) relative to the donor and recipient cell densities, ensuring detection without significant artefacts from mating on transconjugant-selective medium (noise).

**Fig. 4:**
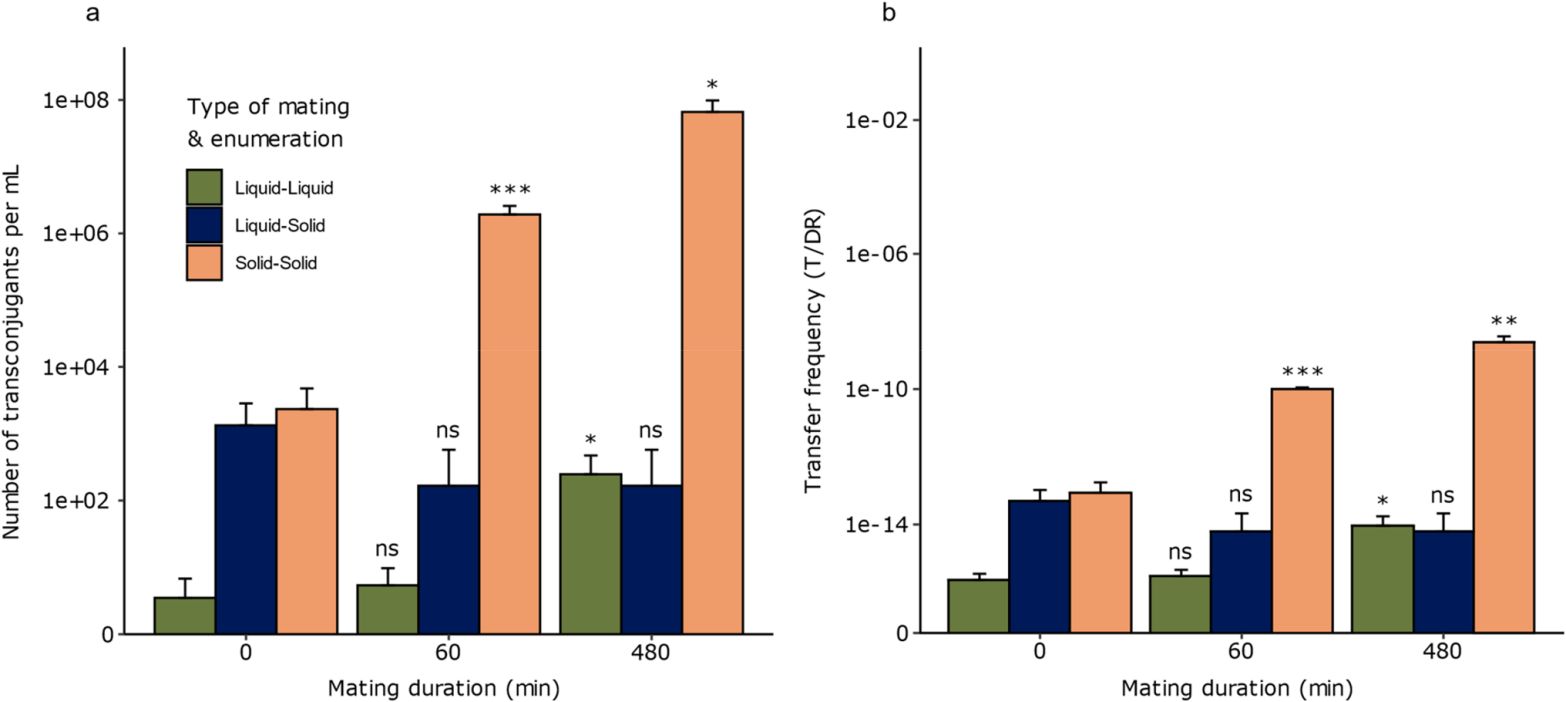
Transconjugant enumeration as a function of mating duration in three mating experiments with inoculation densities ensuring low RP4 transfer on/in transconjugant-selective medium (at 0-min mating control threshold of 28 ± 4 CFU/mm^2^ donors and recipients or below during solid enumeration or 1.68•10^6^ ± 2.46•10^5^ CFU during liquid enumeration). **a** Average concentration of transconjugants per mL of mating mixture estimated on transconjugant-selective medium for different mating durations. Colors represent liquid mating-liquid enumeration (green), liquid mating-solid enumeration (dark blue), and solid mating-solid enumeration (orange). **b** Average apparent conjugation frequencies (T/DR) estimated at each time point, where T, D, and R corresponds to CFU of transconjugants, donors, and recipients per mL. Independent samples t-test was used to compare the mean of the parameters at 0 min against the other time points. Error bars represent standard deviations, and ns and asterisks denote “not significant” and p-values: *p < 0.025, **p < 0.01, ***p < 0.001. Number of replicates for transconjugants, donors and recipients were n = 3 (solid enumeration)/8 (liquid enumeration), 21, and 23, respectively

We first tested the common approach where liquid mating is followed by solid enumeration. However, regardless of the mating duration, this method failed to produce enough transconjugants to exceed the 0-min mating control, which represents mating on transconjugant-selective plates, when plating the density of the 0-min mating control threshold at 28 ± 4 CFU/mm^2^ (Fig. 4a and Tables S6 and S7). These observations indicate that RP4 conjugation frequencies between *E. coli* strains, as estimated from liquid mating, are likely overestimated when determined from CFU plate counting.

Next, we tested solid mating on filters placed on an agar surface followed by solid enumeration, allowing for more efficient mating due to the short, rigid conjugative pili encoded by the RP4 plasmid. Because more transconjugants were formed (higher signal), we observed an increase of transconjugants with mating duration, when plating the density of the 0-min mating control threshold at 28 ± 4 CFU/mm^2^ or lower in contrast to the liquid mating (Fig. 4a and Tables S8 and S9).

Finally, we tested liquid mating followed by liquid enumeration. By using detection in liquid medium where de novo transfer rate is low, it significantly reduced plasmid transfer occurring in the transconjugant-selective medium and resulted in a significant increase in transconjugants after 480 min of mating compared to the 0-min mating control. Hence, this approach increased the signal-to-noise ratio by decreasing the detection noise (Fig. 4a and Tables S10 and S11). The estimated conjugation frequencies (T/DR) calculated by using the CFU counts of transconjugants (T), donors (D), and recipients (R) per mL followed a similar trend (Fig. 4b and Tables S12-S14).

## Discussion

Liquid mating followed by plate counting is a simple and easy method to quantify conjugation frequencies. Despite the relative ease of the method, one essential aspect that is seldom accounted for is the risk of bias introduced by the artefactual increase of transconjugant counts due to plasmid transfer on the transconjugant-selective plates.

Our study confirms that there is a limit to the density of donors and recipients that can be plated to avoid plasmid transfer occurrences on transconjugant-selective plates. For RP4 transfer between *E. coli* strains, if the number of cells plated exceeds 28 ± 4 CFU/mm^2^ (or 1.68•10^5^ ± 2.46•10^4^ CFU/standard 9 cm Petri dish), the estimated conjugation frequency will be largely inflated, irrespective of mating method. Hence, we recommend that for RP4 transfer between *E. coli* strains, the cell density is maintained below this threshold (e.g. by making appropriate dilutions) when enumerating transconjugants via plate counting. A similar finding of a threshold of 10^5^ CFU/Petri dish has been previously reported [21]. Alternatively, by using a liquid enumeration method, we increased the signal-to-noise ratio and managed to successfully enumerate transconjugants without the bias from mating occurring in the selective media. A similar approach has been utilized to detect transconjugants with higher sensitivity compared to traditional plate counting method [32].

We also confirmed that the transfer of RP4 between *E. coli* strains occurs at a very low frequency of approximately 10^–15^ mL/CFU in liquid mating compared to solid mating of approximately 10^–9^ mL/CFU after a mating duration of 480 min. This raises an important question about all the studies that estimate conjugation frequencies of RP4 by a combinational approach of liquid mating and plate count enumeration. Indeed, while controls to check for donors and recipients ability to grow on transconjugant-selective plates by spontaneous mutations are regularly included [36, 40, 41], controls for plasmid transfer on the transconjugant-selective plates are not routinely performed. Therefore, when a new mating assay is developed, we recommend including a 0-min mating control where plating is performed immediately after mixing donors and recipients to identify the dilution range that avoids plasmid transfer on the transconjugant-selective plates. Such a control is also included, as guideline 2a of a recent set of guidelines for the estimation and reporting of plasmid conjugation rates [34].

While our study focused on RP4, one of the most widely used models in plasmid ecology, any plasmid that exhibits significantly higher conjugation efficiency on solid surfaces than in suspension will be at risk of similar spurious inflation of conjugation frequency. This strong preference for transferring on surfaces, compared to liquid transfer, has been observed for diverse plasmids, including IncP-1β plasmid pB10 [42], IncW plasmid R388 [42], IncP-9 plasmid NAH7K2 [42], and Inc18 plasmid pAMβ1 [43] with conjugation frequencies that are several orders of magnitude higher. Additionally, some plasmids, like clinical carbapenemase-encoding plasmids [44], transfer at a low frequency, making it impractical to plate below a certain density threshold. Furthermore, the physiology of the donors and recipients can significantly influence the transfer efficiency. In such scenarios, the general recommendation is to adopt unbiased enumeration methods after conducting mating assays in liquid environments [19, 32, 45–48].

Essentially, estimating plasmid transfer by cultivation on transconjugant-selective plates involves managing the signal-to-noise ratio. The goal is to achieve a high signal from the mating assay (i.e. many transconjugants are generated) relative to the background noise that are transconjugants generated on plates and estimated from the 0-min mating control. Both the signal and the noise are plasmid and assay dependent. For the model plasmid and strains we used, the liquid mating-solid enumeration method has a low signal and high noise, the solid mating-solid enumeration method has high signal and high noise, while the liquid mating-liquid enumeration method has low signal and low noise.

In light of these challenges, the addition of nalidixic acid has been reported to counteract plasmid transfer on transconjugant-selective plates [21]. However, in our study, using nalidixic acid at bactericidal concentration of 50 µg/mL proved insufficient in inhibiting the donors at high cell densities and thus failed to prevent plasmid transfer on transconjugant-selective plates. This is consistent with findings proving that the minimal inhibitory concentration is positively correlated with inoculum size [49]. In any case, a survey of numerous studies estimating conjugation frequencies [11–13, 16, 17, 26, 28, 35, 36, 40, 41, 50] indicates that nalidixic acid is rarely used in transconjugant-selective plates (Tables S3). Instead, combinations of kanamycin, ampicillin, chloramphenicol, streptomycin, tetracycline, and rifampicin are often applied. Moreover, the synergistic and antagonistic behavior of antibiotics on transconjugant-selective plates in the context of transconjugant enumeration is unexplored. It is commonly emphasized that the interaction between bacteriostatic and bactericidal antibiotics results in an antagonistic effect since bacteriostatic drugs antagonize bactericidal drugs that affect dividing cells by inhibiting cell growth [51, 52]. Notable examples of antagonistic effects are interactions between drugs that inhibit the 30S protein synthesis and cell wall synthesis, 50S protein synthesis and DNA replication, as well as folic acid synthesis and cell wall synthesis. In some cases, drug combinations even cause suppression, where one antibiotic alleviates the effect of another [51]. It is therefore conceivable that the combination of tetracycline (bacteriostatic inhibitor of 30S protein synthesis) and ampicillin (bactericidal inhibitor of cell wall synthesis) used in our study may have resulted in an antagonistic drug interaction and diminished the inhibitory effect on the recipients long enough for them to receive plasmids from the donors. Furthermore, the combination of tetracycline and nalidixic acid (bactericidal inhibitor of DNA replication) suggests an antagonistic effect directed toward the donors, with evidence of this occurring in time-kill curves [52].

Overall, mating assays are essential for studying the efficiency of mobilization of genetic elements such as plasmids to gain insight into predicting, hindering, or facilitating plasmid transfer. The main goal with this type of experiment is to accurately estimate the conjugation frequencies.

We demonstrated that plating mixtures of *E. coli* donors harboring the RP4 plasmid and *E. coli* recipients above a certain threshold (28 ± 4 CFU/mm^2^ or 1.68•10^5^ ± 2.46•10^4^ CFU/standard 9 cm Petri dish) artefactually increases transconjugants counts. This increase results from plasmid transfer occurring post-mating on transconjugant-selective plates, ultimately affecting the accuracy of conjugation frequency estimations.

Because plasmids differ in their preferential mating modes (solid surface vs liquid suspension), they can be differentially affected by this problem. For example, for RP4, transconjugants formed in liquid mating assays were undetected because they were lost due to dilution when plating on transconjugant-selective plates at or below the threshold density. Furthermore, this bias can also be affected by the type of strain, the type of stresses (oxygen, carbon, and nitrogen limitation) that are introduced during the mating assay, and the antibiotics used for the transconjugant-selective plates (type and concentration).

Therefore, as a final remark, when working with *E. coli* strains and the RP4 plasmid, we recommend plating below the cell density threshold and/or performing additional controls where donors and recipients are briefly mixed before plating at the same dilutions as for the actual mating. Alternatively, adopting a robust and unbiased enumeration method can help prevent artefactual increases of transconjugants. It is important to note that a universal plating threshold cannot be derived from this study due to the complex interplay between plasmid transfer time and antibiotic action. To address this gap, further research is needed, incorporating a wider range of strains and plasmids, as well as varying types and concentrations of antibiotics, to provide a more comprehensive understanding of the factors influencing plating thresholds.

### Supplementary Information

Below is the link to the electronic supplementary material.

Supplementary file1 (DOCX 2204 KB)

## Data Availability

No datasets were generated or analysed during the current study.
