# Supplementary material for: Mating Assay: Plating Below a Cell Density Threshold is Required for Unbiased Estimation of Plasmid Conjugation Frequency of RP4 Transfer Between E. coli Strains
Source: Microb Ecol. 2024 Aug 28;87:109. doi: 10.1007/s00248-024-02427-7 (PMC11358341; doi:10.1007/s00248-024-02427-7)
Supplement: Supplementary file 1 [file 248_2024_2427_MOESM1_ESM.docx]

**Supplementary information**

Mating assay: Plating below a cell density threshold is required for unbiased estimation of plasmid conjugation frequency of RP4 transfer between *E. coli* strains

Microbial Ecology

*Zhiming He^a,b*^, Barth F. Smets^c^, Arnaud Dechesne^a*^*

^a^Department of Biotechnology and Biomedicine, Technical University of Denmark, Søltofts Plads Building 221, 2800 Kgs. Lyngby, Denmark

^b^Sino-Danish College (SDC) for Education and Research, University of Chinese Academy of Sciences, 8000 Aarhus C, Denmark

^c^Department of Biological and Chemical Engineering – Environmental Engineering, Aarhus University, Ole Worms Allé 3, 8000 Aarhus C, Denmark

**^*^Corresponding author information**

Email: [zhihe@dtu.dk](mailto:zhihe@dtu.dk)

Email: [arde@dtu.dk](mailto:arde@dtu.dk)

16 pages; 14 Tables and 3 Figures

**Example of calculation:**

(1) **MPN/g =** ${\sum\boldsymbol{g}_{\boldsymbol{j}}\boldsymbol{/(}\sum\boldsymbol{t}_{\boldsymbol{j}}\boldsymbol{m}_{\boldsymbol{j}}\sum\boldsymbol{(}\boldsymbol{t}_{\boldsymbol{j}}\boldsymbol{-}\boldsymbol{g}_{\boldsymbol{j}}\boldsymbol{)}\boldsymbol{m}_{\boldsymbol{j}}\boldsymbol{)}}^{\boldsymbol{(}\frac{\boldsymbol{1}}{\boldsymbol{2}}\boldsymbol{)}}$	MPN = CFU & g = mL
(2) $\sum\boldsymbol{g}_{\boldsymbol{j}}$	= 37 + 15 **= 52**
(3) $\sum\boldsymbol{t}_{\boldsymbol{j}}\boldsymbol{m}_{\boldsymbol{j}}$	**=** 48 * 0.01 + 48 * 0.001 **= 0.528**
(4) $\sum\boldsymbol{(}\boldsymbol{t}_{\boldsymbol{j}}\boldsymbol{-}\boldsymbol{g}_{\boldsymbol{j}}\boldsymbol{)}\boldsymbol{m}_{\boldsymbol{j}}$	**=** 11 * 0.01 + 33 * 0.001 **= 0.143**
(5) ${\mathbf{52}\mathbf{/(}\mathbf{0}\mathbf{.}\mathbf{528}\mathbf{*}\mathbf{0}\mathbf{.}\mathbf{143}\mathbf{)}}^{\boldsymbol{(}\frac{\boldsymbol{1}}{\boldsymbol{2}}\boldsymbol{)}}$	**= 189** CFU/mL
(6) [Most Probable Number Calculator (foodsafetyrisk.org)](https://pub-connect.foodsafetyrisk.org/microbial/mpncalc/)

**Fig. S1** Overview of the most-probable-number liquid enumeration method. Mating mixture is added to a 96-well microplate undiluted (UD) and at a ten-fold dilution (10x). A total of 48 aliquots of 10 µL is added for each dilution. (1) shows the Thomas formula to calculate the original concentration in the mating mixture[1]. (2) denotes the number of positive wells in the selected dilutions where 37/48 (UD) and 15/48 (10x) are shown in the illustration above. (3) denotes the grams of sample in all wells in the selected dilutions where the original sample represents 1 gram while UD is 0.01 gram and 10x is 0.001 gram. (4) denotes the grams of sample in all negative wells in the selected dilutions where the original sample represents 1 gram while UD is 0.01 gram and 10x is 0.001 gram. (5) using the formula in (1) with the numbers calculated in (2)-(4), the cell concentration can be estimated. (6) directs to a web-based calculator (coded with knowledge from [1]) that can be used for calculating the most-probable-number in an easy and intuitive manner

**Fig. S2** Illustrating the random distribution of Poisson-distributed points across space. Each point represents either a donor (blue) or a recipient (red) cell randomly placed on a two-dimensional area of 1 mm^2^ with cell densities of (a) 3 (b) 28 ± 4, (c) 277 ± 40, and (d) 2769 ± 405 CFU/mm^2^. A crucial metric to consider is the likelihood of encountering cells at a distance equal to or less than the length of the conjugative pilus. At such proximity, it is under this assumption that cell-to-cell contact between donor and recipient cells is expected. This facilitates the process of conjugation and generates a new transconjugant., which can be plotted at different cell densities (λ). While no concrete evidence exists about the length of the RP4 conjugative pilus, it is known that the P-like pili are short, rigid, and thin. Previously, 779 out of the 855 successful mating pairs found on filters occurred through direct cell-to-cell contact at the 0-1 µm range for a IncP-9 plasmid[2]. It has also been mentioned that P-like pili are usually less than 1 µm in length[3]

**Fig. S3** Frequency distribution of the distances to the nearest neighboring cells for each individual simulated Poisson-distributed point representing either a donor or recipient cell across a two-dimensional space with an area of 1 mm^2^. The cell densities are (a) 28 ± 4 (b) 277 ± 40, and (c) 2769 ± 405 CFU/mm^2^

**Table. S1** Overview of the model bacterial strains and conjugative plasmid

| Bacterium | Strain | Chromosomal markers | Plasmid | Role | Source |
| --- | --- | --- | --- | --- | --- |
| *E. coli* | MG1655 | - | RP4 | Donor | This study |
| *E. coli* | MG1655 | Kan^R^, Nal^R^, Rif^R^ | - | Recipient | Søren Sørensen’s lab (Department of Biology KU) |

| Plasmid | Size (kbp) | Plasmid markers | Inc group | Host range | Copy number | Source |
| --- | --- | --- | --- | --- | --- | --- |
| RP4 | Ca. 60 | gfp, Tet^R^, Amp^R^, Kan^R^ | IncPα | Broad | Low | Søren Sørensen’s lab (Department of Biology KU) |

**Table. S2** Relation between optical density and cell density (CFU/mL).

| **Strain** | **OD600 measurement** | **Raw data** | | | **Avg. CFU/mL** |
| --- | --- | --- | --- | --- | --- |
|  |  | **Dilution** | **Volume plated/spotted** | **CFU** |  |
| *E. coli* MG1655 (RP4) | ~ 0.5 | 10^-5^ | 10 µL | 24, 28, 27 | 2.02•10^8^ ± 8.24•10^7^ |
|  |  |  |  | 26, 32, 19 |  |
|  |  |  |  | 22, 24, 34 |  |
|  |  |  |  | 28, 19, 19 |  |
|  |  |  |  | 25, 37, 16 |  |
|  |  |  |  | 33, 31, 18 |  |
|  |  |  |  | 30, 29, 39 |  |
|  |  |  |  | 36, 43, 49 |  |
|  |  |  | 100 µL | 140, 175, 139, 110 |  |
|  |  |  |  | 70, 120, 104, 106 |  |
|  |  |  |  | 129, 134, 145 |  |
|  |  |  |  | 251, 190, 112 |  |
|  |  |  |  | 127, 105, 70, 105 |  |
|  |  |  |  | 163, 112, 78, 123 |  |
|  |  |  |  | 203, 186, 64, 116 |  |
|  |  |  |  | 144, 143, 168, 183, 153, 184 |  |
|  |  |  |  | 182, 81, 66, 97, 136, 129 |  |
|  |  |  |  | 158, 167, 173, 181, 163, 146 |  |
|  |  |  |  | 183, 187, 192, 225, 224, 170 |  |
|  |  |  |  | 233, 162, 164, 189, 207, 198 |  |
|  |  |  |  | 201, 222, 191, 189, 182, 151 |  |

| *E. coli* MG1655-Nal^R^-Rif^R^-Kan^R^ | ~ 0.5 | 10^-5^ | 10 µL | 31, 32, 20 | 1.35•10^8^ ± 8.41•10^7^ |
| --- | --- | --- | --- | --- | --- |
|  |  |  |  | 21, 20, 22 |  |
|  |  |  |  | 28,28, 25 |  |
|  |  |  |  | 20, 20, 22 |  |
|  |  |  |  | 21, 23, 13 |  |
|  |  |  |  | 20, 13, 19 |  |
|  |  |  |  | 26, 31, 20 |  |
|  |  |  |  | 24, 29, 30 |  |
|  |  |  |  | 8, 10, 8, 19 |  |
|  |  |  |  | 15, 27, 21, 17 |  |
|  |  |  | 100 µL | 49, 82, 67, 30 |  |
|  |  |  |  | 82, 65, 76, 33 |  |
|  |  |  |  | 51, 72, 113 |  |
|  |  |  |  | 71, 25, 26 |  |
|  |  |  |  | 48, 32, 59, 50 |  |
|  |  |  |  | 40, 32, 29, 68 |  |
|  |  |  |  | 26, 28, 22, 18 |  |
|  |  |  |  | 121, 87, 95, 102, 116, 88 |  |
|  |  |  |  | 146, 85, 62, 80, 85, 100 |  |
|  |  |  |  | 69, 89, 30, 63, 69, 70 |  |
|  |  |  |  | 108, 146, 104, 107, 118, 102 |  |
|  |  |  |  | 102, 104, 66, 94, 141, 114 |  |
|  |  |  |  | 129, 101, 99, 67, 88, 120 |  |

**Table. S3** Overview of commonly used antibiotics, their mechanism and mode of action, and drug combinations found in other studies

| Antibiotics | Mechanism of action | Mode of action |
| --- | --- | --- |
| Kanamycin (Kan) | Inhibits 30S protein synthesis | Bactericidal |
| Ampicillin (Amp) | Inhibits cell wall synthesis | Bactericidal |
| Streptomycin (Str) | Inhibits 30S protein synthesis | Bactericidal |
| Nalidixic acid (Nal) | Inhibits DNA replication | Bactericidal |
| Chloramphenicol (Chl) | Inhibits 50S protein synthesis | Bacteriostatic |
| Tetracycline (Tet) | Inhibits 30S protein synthesis | Bacteriostatic |
| Rifampicin (Rif) | Inhibits DNA dependent RNA pol. | Bacteriostatic |
| Sulfamethoxazole (Smx) | Inhibits purine synthesis | Bacteriostatic |
| Trimethoprim (Tmp) | Inhibits folic acid synthesis | Bacteriostatic (or –cidal when combined with Sulfonamides) |

| Drug combination on transconjugant-selective plates | References |
| --- | --- |
| Tet, Amp, Nal | This study |
| Kan, Chl | [4] |
| Kan, Chl, Tet, Amp | [5] |
| Kan, Chl, Tet, Amp | [6] |
| Kan, Chl, Tet, Amp | [7] |
| Kan, Chl, Amp | [8] |
| Kan, Str | [9] |
| Kan, Chl, Tet, Amp | [10] |
| Kan, Str, Tet, Amp | [11] |
| Kan, Rif, Tet, Amp | [12] |
| Kan, Chl | [13] |
| Kan, Chl, Tet, Amp | [14] |
| Kan, Str, Tet, Amp | [15] |
| Rif, Amp | [16] |
| Kan, Chl | [17] |
| Rif, Amp | [18] |
| Kan, Chl | [19] |
| Rif, Tmp | [20] |
| Kan, Str, Tet, Amp | [21] |
| Kan, Chl, Amp | [22] |
| Kan, Chl, Tet, Amp | [23] |
| Kan, Amp | [24] |
| Tet, Amp, Rif | [25] |
| Kan, Rif, Tet, Amp | [26] |
| Kan. Rif, Smx | [27] |
| Kan, Str, Tet, Amp | [28] |
| Rif, Amp | [29] |
| Rif, Tet, Nal | [30] |

**Table. S4** Variables to be measured in the laboratory

| **Required measurements** | **Method** | **Description** |
| --- | --- | --- |
| Transconjugant (T) | T/DR | Final transconjugant density |
| Donor (D) |  | Initial donor density |
| Recipient (R) |  | Initial recipient density |

**Table. S5** Expected outcomes in the chosen selective conditions

| **Mating assay** | **Quantification**  **Method** | **Required**  **measurements** | **Description of selection** | | | **Expected selective outcomes** | | |
| --- | --- | --- | --- | --- | --- | --- | --- | --- |
|  |  |  | **Selective agent(s)** | **Purpose** | **Media agar** | **Donor**  **(D)** | **Recipient (R)** | **Transconjugant (T)** |
| Liquid-Solid | T/DR | T, D, and R | Tet, Amp | Selection for D | Agar | Colony formation | No colony formation | Colony formation |
| Liquid-Solid |  |  | Nal | Selection for R | Agar | No colony formation | Colony formation | Colony formation |
| Liquid-Solid |  |  | Tet, Amp, Nal | Selection for T | Agar | No colony formation | No colony formation | Colony formation |
| Solid-Solid |  |  | Tet, Amp | Selection for D | Agar | Colony formation | No colony formation | Colony formation |
| Solid-Solid |  |  | Nal | Selection for R | Agar | No colony formation | Colony formation | Colony formation |
| Solid-Solid |  |  | Tet, Amp, Nal | Selection for T | Agar | No colony formation | No colony formation | Colony formation |
| Liquid-Liquid |  |  | Tet, Amp | Selection for D | Liquid | Turbid culture | Non-turbid culture | Turbid culture |
| Liquid-Liquid |  |  | Nal | Selection for R | Liquid | Non-turbid culture | Turbid culture | Turbid culture |
| Liquid-Liquid |  |  | Tet, Amp, Nal | Selection for T | Liquid | Non-turbid culture | Non-turbid culture | Turbid culture |

**Table. S6** Raw data of average CFU counts of transconjugants enumerated by plating 100 µL of different dilutions on selective plates at different timepoints (0, 60, and 480 min) after by liquid mating at cell density of 1.68•10^8^ ± 2.46•10^7^ CFU/mL of donors and recipients

| **Density of cells** | | **Mating time (min)** | | |
| --- | --- | --- | --- | --- |
| **In liquid mating (CFU/mL)** | **On selective plates (CFU/mm^2^)** | **0** | **60** | **480** |
| 1.68•10^8^ ± 2.46•10^7^ | 2768 ± 405 | TNTC^a^ | TNTC^a^ | TNTC^a^ |
|  | 277 ± 40 | 58.5 ± 15.4 | 44 ± 8.44 | 1.83 ± 1.83^b^ |
|  | 28 ± 4 | 1.33 ± 1.51 | 0.16 ± 0.4 | 0 |
|  | 3 | 0.16 ± 0.4 | 0 | 0 |

^a^: Too numerous to count, ^b^: Experimental mistake during plating

**Table. S7** Raw data of CFU counts of transconjugants (2 technical replicates and 3 biological replicates) enumerated by plating 100 µL of different dilutions on selective plates at different timepoints (0, 60, and 480 min) after liquid mating at cell density of 1.68•10^8^ ± 2.46•10^7^ CFU/mL of donors and recipients

|  | | **Mating time (min)** | | |
| --- | --- | --- | --- | --- |
| **On selective plates (CFU/mm^2^)** | **Volume plated** | **0** | **60** | **480** |
| 2768 ± 405 | 100 µl | TNTC^a^x2  TNTC^a^x2  TNTC^a^x2 | TNTC^a^x2  TNTC^a^x2  TNTC^a^x2 | TNTC^a^x2  TNTC^a^x2  TNTC^a^x2 |
| 277 ± 40 |  | 39, 61  73, 52  47, 79 | 49, 55  49, 35  42, 34 | 1, 0^b^  3, 5^b^  1, 1^b^ |
| 28 ± 4 |  | 1, 2  4, 0  0, 1 | 0, 0  0, 0  0, 1 | 0, 0  0, 0  0, 1 |
| 3 |  | 1, 0  0, 0  0, 0 | 0, 0  0, 0  0, 0 | 1, 0  0, 0  0, 0 |

^a^: Too numerous to count, ^b^: Experimental mistake during plating

**Table. S8** Raw data of average CFU counts of transconjugants enumerated by plating 100 µL of different dilutions on selective plates at different timepoints (0, 60, and 480 min) after solid mating using cell densities of 1.68•10^5^ ± 2.46•10^4^ CFU/mm^2^ of donors and recipients

| **Density of cells** | | **Mating time (min)** | | |
| --- | --- | --- | --- | --- |
| **On filter mating (CFU/mm^2^)** | **On selective plates (CFU/mm^2^)** | **0** | **60** | **480** |
| 1.68•10^5^ ± 2.46•10^4^ | 2768 ± 405 | TNTC^a^ | TNTC^a^ | TNTC^a^ |
|  | 277 ± 40 | 72.5 ± 15.9 | TNTC^a^ | TNTC^a^ |
|  | 28 ± 4 | 2.33 ± 2.42 | TNTC^a^ | TNTC^a^ |
|  | 3 | 0 | 284.5 ± 18.7 | TNTC^a^ |
|  | <1 | 0 | 19.16 ± 6.65 | TNTC^a^ |
|  | <<1 | NA^b^ | NA^b^ | 66.16 ± 32.1 |

^a^: Too numerous to count, ^b^: Not available

**Table. S9** Raw data of CFU counts of transconjugants (2 technical replicates and 3 biological replicates) enumerated by plating 100 µL of different dilutions on selective plates at different timepoints (0, 60, and 480 min) after liquid mating at cell density 1.68•10^5^ ± 2.46•10^4^ CFU/mm^2^ of donors and recipients

|  | | **Mating time (min)** | | |
| --- | --- | --- | --- | --- |
| **On selective plates (CFU/mm^2^)** | **Volume plated** | **0** | **60** | **480** |
| 2768 ± 405 | 100 µl | TNTC^a^x2  TNTC^a^x2  TNTC^a^x2 | TNTC^a^x2  TNTC^a^x2  TNTC^a^x2 | TNTC^a^x2  TNTC^a^x2  TNTC^a^x2 |
| 277 ± 40 |  | 95, 73  63, 83  49, 72 | TNTC^a^x2  TNTC^a^x2  TNTC^a^x2 | TNTC^a^x2  TNTC^a^x2  TNTC^a^x2 |
| 28 ± 4 |  | 0, 1  6, 4  3, 0 | TNTC^a^x2  TNTC^a^x2  TNTC^a^x2 | TNTC^a^x2  TNTC^a^x2  TNTC^a^x2 |
| 3 |  | 0, 0  0, 0  0, 0 | 262, 261  291, 300  289, 304 | TNTC^a^x2  TNTC^a^x2  TNTC^a^x2 |
| <1 |  | 0, 0  0, 0  0, 0 | 23, 15  29, 11  15, 22 | TNTC^a^x2  TNTC^a^x2  TNTC^a^x2 |
| <<1 |  | NA^b^  NA^b^  NA^b^ | NA^b^  NA^b^  NA^b^ | 13, 53  99, 98  72, 62 |

^a^: Too numerous to count, ^b^: Not available

**Table. S10** Raw data of average CFU of transconjugants enumerated by diluting in selective medium at different timepoints (0, 60, and 480 min) after liquid mating at cell density of 1.68•10^8^ ± 2.46•10^7^ CFU/mL of donors and recipients

| **Density of cells** | | **Mating time (min)** | | |
| --- | --- | --- | --- | --- |
| **In liquid mating (CFU/mL)** | **In selective medium (CFU)** | **0** | **60** | **480** |
| 1.68•10^8^ ± 2.46•10^7^ | 1.68•10^6^ ± 2.46•10^5^  &  1.68•10^5^ ± 2.46•10^4^ | 3.5 ± 3.28 | 5.4 ± 4.32 | 247.25 ± 224.61 |

**Table. S11** Raw data of CFU counts of transconjugants (8 biological replicates) enumerated by inoculating 10 µL of different dilutions in selective medium at different timepoints (0, 60, and 480 min) after liquid mating at cell density of 1.68•10^8^ ± 2.46•10^7^ CFU/mL of donors and recipients

|  | | **Mating time (min)** | | |
| --- | --- | --- | --- | --- |
| **In liquid mating (CFU/mL)** | **In selective medium (CFU)** | **0** | **60** | **480** |
| 1.68•10^8^ ± 2.46•10^7^ | 1.68•10^6^ ± 2.46•10^5^  &  1.68•10^5^ ± 2.46•10^4^ | 1.05, 11, 2,1, 2.1, 2.1, 4.3, 4.3, 1.05 | 9.9, 9.9, 9.9, 0.95, 0.95, 1.9, 1.9, 7.8 | 38, 190, 150, 67, 410, 460, 33, 630 |

**Table. S12** Estimated transfer frequencies (T/DR) at different timepoints (0, 60, and 480 min) after liquid mating followed by solid enumeration at cell density of 1.68•10^8^ ± 2.46•10^7^ CFU/mL of donors and recipients

| **Density of cells** | | **Mating time (min)** | | |
| --- | --- | --- | --- | --- |
| **In liquid mating (CFU/mL)** | **On selective plates (CFU/mm^2^)** | **0** | **60** | **480** |
| 1.68•10^8^ ± 2.46•10^7^ | 2768 ± 405 | NA^a^ | NA^a^ | NA^a^ |
|  | 277 ± 40 | 2.15•10^-13^  ±  5.67•10^-14^ | 1.62•10^-13^  ±  3.10•10^-14^ | 6.73•10^-15b^  ±  6.74•10^-15b^ |
|  | 28 ± 4 | 4.89•10^-14^  ±  5.53•10^-14^ | 6.12•10^-15^  ±  1.5•10^-14^ | 6.12•10^-15^  ±  1.5•10^-14^ |
|  | 3 | 6.12•10^-14^  ±  1.50•10^-13^ | 0 | 6.12•10^-14^  ±  1.50•10^-13^ |

^a^:Not available, ^b^:Experimental mistake during plating

**Table. S13** Estimated transfer frequencies (T/DR) at different timepoints (0, 60, and 480 min) after solid mating followed by solid enumeration at cell density of 1.68•10^5^ ± 2.46•10^4^ CFU/mm^2^ of donors and recipients

| **Density of cells** | | **Mating time (min)** | | |
| --- | --- | --- | --- | --- |
| **On filter mating (CFU/mm^2^)** | **On selective plates (CFU/mm^2^)** | **0** | **60** | **480** |
| 1.68•10^5^ ± 2.46•10^4^ | 2768 ± 405 | NA^a^ | NA^a^ | NA^a^ |
|  | 277 ± 40 | 2.66•10^-13^  ±  5.83•10^-14^ | NA^a^ | NA^a^ |
|  | 28 ± 4 | 8.57•10^-14^  ±  8.89•10^-14^ | NA^a^ | NA^a^ |
|  | 3 | 0 | 1.04•10^-10^  ±  6.85•10^-12^ | NA^a^ |
|  | <1 | 0 | 7.04•10^-11^  ±  2.44•10^-11^ | NA^a^ |
|  | <<1 | NA^a^ | NA^a^ | 2.43•10^-9^  ±  1.18•10^-9^ |

^a^:Not available

**Table. S14** Estimated transfer frequencies (T/DR) at different timepoints (0, 60, and 480 min) after liquid mating followed by liquid enumeration at cell density of 1.68•10^8^ ± 2.46•10^7^ CFU/mL of donors and recipients

| **Density of cells** | | **Mating time (min)** | | |
| --- | --- | --- | --- | --- |
| **In liquid mating (CFU/mL)** | **In selective medium (CFU)** | **0** | **60** | **480** |
| 1.68•10^8^ ± 2.46•10^7^ | 1.68•10^6^ ± 2.46•10^5^  &  1.68•10^5^ ± 2.46•10^4^ | 1.29•10^-16^  ±  1.21•10^-16^ | 1.98•10^-16^  ±  1.59•10^-16^ | 9.08•10^-15^  ±  8.25•10^-15^ |

**References**

[1]	R. Blodgett, “BAM Appendix 2: Most Probable Number from Serial Dilutions,” U.S Food & Drug Administration. Accessed: Jul. 25, 2024. [Online]. Available: https://www.fda.gov/food/laboratory-methods-food/bam-appendix-2-most-probable-number-serial-dilutions#background

[2]	J. Seoane, T. Yankelevich, A. Dechesne, B. Merkey, C. Sternberg, and B. F. Smets, “An individual-based approach to explain plasmid invasion in bacterial populations,” *FEMS Microbiol. Ecol.*, vol. 75, no. 1, pp. 17–27, 2011, doi: 10.1111/j.1574-6941.2010.00994.x.

[3]	T. D. Lawley, W. A. Klimke, M. J. Gubbins, and L. S. Frost, “F factor conjugation is a true type IV secretion system,” *FEMS Microbiol. Lett.*, vol. 224, no. 1, pp. 1–15, 2003, doi: 10.1016/S0378-1097(03)00430-0.

[4]	Y. Zhang, A. Z. Gu, M. He, D. Li, and J. Chen, “Subinhibitory Concentrations of Disinfectants Promote the Horizontal Transfer of Multidrug Resistance Genes within and across Genera,” *Environ. Sci. Technol.*, vol. 51, no. 1, pp. 570–580, 2017, doi: 10.1021/acs.est.6b03132.

[5]	Z. Yu, Y. Wang, J. Lu, P. L. Bond, and J. Guo, “Nonnutritive sweeteners can promote the dissemination of antibiotic resistance through conjugative gene transfer,” *ISME J.*, vol. 15, no. 7, pp. 2117–2130, 2021, doi: 10.1038/s41396-021-00909-x.

[6]	Y. Wang *et al.*, “Non-antibiotic pharmaceuticals promote the transmission of multidrug resistance plasmids through intra- and intergenera conjugation,” *ISME J.*, vol. 15, no. 9, pp. 2493–2508, 2021, doi: 10.1038/s41396-021-00945-7.

[7]	J. Lu, Y. Wang, M. Jin, Z. Yuan, P. Bond, and J. Guo, “Both silver ions and silver nanoparticles facilitate the horizontal transfer of plasmid-mediated antibiotic resistance genes,” *Water Res.*, vol. 169, 2020, doi: 10.1016/j.watres.2019.115229.

[8]	J. Lu *et al.*, “Triclosan at environmentally relevant concentrations promotes horizontal transfer of multidrug resistance genes within and across bacterial genera,” *Environ. Int.*, vol. 121, no. October, pp. 1217–1226, 2018, doi: 10.1016/j.envint.2018.10.040.

[9]	Z. Qiu *et al.*, “Nanoalumina promotes the horizontal transfer of multiresistance genes mediated by plasmids across genera,” *Proc. Natl. Acad. Sci. U. S. A.*, vol. 109, no. 13, pp. 4944–4949, 2012, doi: 10.1073/pnas.1107254109.

[10]	S. Zhang, Y. Wang, H. Song, J. Lu, Z. Yuan, and J. Guo, “Copper nanoparticles and copper ions promote horizontal transfer of plasmid-mediated multi-antibiotic resistance genes across bacterial genera,” *Environ. Int.*, vol. 129, no. May, pp. 478–487, 2019, doi: 10.1016/j.envint.2019.05.054.

[11]	X. Wang *et al.*, “Bacterial exposure to ZnO nanoparticles facilitates horizontal transfer of antibiotic resistance genes,” *NanoImpact*, vol. 10, no. November 2017, pp. 61–67, 2018, doi: 10.1016/j.impact.2017.11.006.

[12]	Z. Qiu *et al.*, “Effects of nano-TiO2 on antibiotic resistance transfer mediated by RP4 plasmid,” *Nanotoxicology*, vol. 9, no. 7, pp. 895–904, 2015, doi: 10.3109/17435390.2014.991429.

[13]	Y. Zhang *et al.*, “Sub-inhibitory concentrations of heavy metals facilitate the horizontal transfer of plasmid-mediated antibiotic resistance genes in water environment,” *Environ. Pollut.*, vol. 237, pp. 74–82, 2018, doi: 10.1016/j.envpol.2018.01.032.

[14]	Y. Wang *et al.*, “Antiepileptic drug carbamazepine promotes horizontal transfer of plasmid-borne multi-antibiotic resistance genes within and across bacterial genera,” *ISME J.*, vol. 13, no. 2, pp. 509–522, 2019, doi: 10.1038/s41396-018-0275-x.

[15]	Q. Wang, D. Mao, and Y. Luo, “Ionic Liquid Facilitates the Conjugative Transfer of Antibiotic Resistance Genes Mediated by Plasmid RP4,” *Environ. Sci. Technol.*, vol. 49, no. 14, pp. 8731–8740, 2015, doi: 10.1021/acs.est.5b01129.

[16]	B. Yang, Z. Wang, Y. Jia, D. Fang, R. Li, and Y. Liu, “Paclitaxel and its derivative facilitate the transmission of plasmid-mediated antibiotic resistance genes through conjugative transfer,” *Sci. Total Environ.*, vol. 810, p. 152245, Mar. 2022, doi: 10.1016/j.scitotenv.2021.152245.

[17]	T. Cen, X. Zhang, S. Xie, and D. Li, “Preservatives accelerate the horizontal transfer of plasmid-mediated antimicrobial resistance genes via differential mechanisms,” *Environ. Int.*, vol. 138, no. January, p. 105544, 2020, doi: 10.1016/j.envint.2020.105544.

[18]	Y. Jia, Z. Wang, D. Fang, B. Yang, R. Li, and Y. Liu, “Acetaminophen promotes horizontal transfer of plasmid-borne multiple antibiotic resistance genes,” *Sci. Total Environ.*, vol. 782, p. 146916, 2021, doi: 10.1016/j.scitotenv.2021.146916.

[19]	Y. Zhang, A. Z. Gu, T. Cen, X. Li, D. Li, and J. Chen, “Petrol and diesel exhaust particles accelerate the horizontal transfer of plasmid-mediated antimicrobial resistance genes,” *Environ. Int.*, vol. 114, no. February, pp. 280–287, 2018, doi: 10.1016/j.envint.2018.02.038.

[20]	S. B. I. Schmidt, A. Rodríguez-Rojas, J. Rolff, and F. Schreiber, “Biocides Used as Material Preservatives Modify Rates of de novo Mutation and Horizontal Gene Transfer in Bacteria,” *J. Hazard. Mater.*, vol. 437, no. May, p. 129280, 2022, doi: 10.1016/j.jhazmat.2022.129280.

[21]	H. Huang *et al.*, “Nitric Oxide: A Neglected Driver for the Conjugative Transfer of Antibiotic Resistance Genes among Wastewater Microbiota,” *Environ. Sci. Technol.*, 2022, doi: 10.1021/acs.est.2c01889.

[22]	P. Ding, J. Lu, Y. Wang, M. A. Schembri, and J. Guo, “Antidepressants promote the spread of antibiotic resistance via horizontally conjugative gene transfer,” *Environ. Microbiol.*, vol. 24, no. 11, pp. 5261–5276, 2022, doi: 10.1111/1462-2920.16165.

[23]	H. Zhang, J. Liu, L. Wang, and Z. Zhai, “Glyphosate escalates horizontal transfer of conjugative plasmid harboring antibiotic resistance genes,” *Bioengineered*, vol. 12, no. 1, pp. 63–69, 2021, doi: 10.1080/21655979.2020.1862995.

[24]	H. Tang, Z. Liu, B. Hu, and L. Zhu, “Effects of iron mineral adhesion on bacterial conjugation: Interfering the transmission of antibiotic resistance genes through an interfacial process,” *J. Hazard. Mater.*, vol. 435, no. April, p. 128889, 2022, doi: 10.1016/j.jhazmat.2022.128889.

[25]	W. Li *et al.*, “Environmentally relevant concentrations of mercury facilitate the horizontal transfer of plasmid-mediated antibiotic resistance genes,” *Sci. Total Environ.*, vol. 852, no. July, p. 158272, 2022, doi: 10.1016/j.scitotenv.2022.158272.

[26]	K. He *et al.*, “Low-concentration of trichloromethane and dichloroacetonitrile promote the plasmid-mediated horizontal transfer of antibiotic resistance genes,” *J. Hazard. Mater.*, vol. 425, p. 128030, 2022, doi: 10.1016/j.jhazmat.2021.128030.

[27]	J. Jutkina, N. P. Marathe, C. F. Flach, and D. G. J. Larsson, “Antibiotics and common antibacterial biocides stimulate horizontal transfer of resistance at low concentrations,” *Sci. Total Environ.*, vol. 616–617, pp. 172–178, 2018, doi: 10.1016/j.scitotenv.2017.10.312.

[28]	J. Liao, H. Huang, and Y. Chen, “CO2 promotes the conjugative transfer of multiresistance genes by facilitating cellular contact and plasmid transfer,” *Environ. Int.*, vol. 129, no. April, pp. 333–342, 2019, doi: 10.1016/j.envint.2019.05.060.

[29]	T. S. B. Møller *et al.*, “Treatment with cefotaxime affects expression of conjugation associated proteins and conjugation transfer frequency of an IncI1 plasmid in Escherichia coli,” *Front. Microbiol.*, vol. 8, no. NOV, pp. 1–9, 2017, doi: 10.3389/fmicb.2017.02365.

[30]	M. T. Guo, Q. Bin Yuan, and J. Yang, “Distinguishing effects of ultraviolet exposure and chlorination on the horizontal transfer of antibiotic resistance genes in municipal wastewater,” *Environ. Sci. Technol.*, vol. 49, no. 9, pp. 5771–5778, 2015, doi: 10.1021/acs.est.5b00644.
